# Estimating time-related decline from mathematics platform logs: rapid repetition, learner variation, and model-implied review intervals

**DOI:** 10.3389/fpsyg.2026.1908596

**Published:** 2026-10-28

**Authors:** Yang Liu, Nurzatulshima Kamarudin, Tajularipin Sulaiman

**Affiliations:** 1 Institute for Mathematical Research, Universiti Putra Malaysia, Serdang, Selangor Darul Ehsan, Malaysia; 2 School of Mathematics and Big Data, Neijiang Normal University, Neijiang, China; 3 Faculty of Educational Studies, Universiti Putra Malaysia, Serdang, Selangor Darul Ehsan, Malaysia

**Keywords:** forgetting curve, knowledge tracing, learning analytics, mathematics education, personalized learning, retention probability, spaced repetition

## Abstract

**Introduction:**

Digital mathematics platforms record repeated encounters with previously practiced knowledge, but these records combine rapid repetitions with retrieval after longer delays. This study examined how different revisit intervals affect estimates of time-related decline in response correctness and the review intervals derived from these estimates.

**Methods:**

We analyzed repeated learner–knowledge component (KC) encounters in the public Junyi Academy Online Learning Activity Dataset. Exponential, power-law, and hyperbolic retention curves were compared using revisits separated by at least 1 h. A generalized linear model estimated the association between elapsed time and response correctness while adjusting for prior encounters, previous correctness, and KC differences. Learner-level decay estimates were used to calculate model-implied review intervals. A sensitivity analysis further examined learner-specific time slopes after accounting for practice history, previous correctness, KC effects, and learner baselines, with predictive performance evaluated on later responses.

**Results:**

When all positive revisit delays were included, each additional day was associated with almost no change in the odds of a correct response (odds ratio = 0.9973). The estimated decline became more pronounced after rapid repetitions were excluded, reaching an odds ratio of 0.9706 for intervals of at least 1 day. Exponential and hyperbolic curves showed nearly equivalent fits for revisits separated by at least 1 h. Unadjusted learner-level estimates yielded a median model-implied review interval of 6.21 days, with an interquartile range of 2.12-16.66 days. After adjustment, learner-specific time slopes were weakly associated with the unadjusted decay estimates and did not improve prediction of subsequent responses.

**Discussion:**

Rapid repetitions substantially influence estimates of time-related decline derived from mathematics platform logs. Although learner-level retention curves can be used to calculate review intervals, these intervals remain model-derived estimates rather than validated scheduling recommendations. Further learner–KC-level estimation and prospective validation are needed to determine whether the proposed intervals can reliably support personalized review scheduling.

## 1 Introduction

Digital mathematics platforms turn response histories into estimates of mastery, readiness, and likely success on a subsequent problem. These estimates support the adaptation of content, pacing, and feedback (Farhood et al., 2025; Hariyanto et al., 2025; Tan et al., 2025; Khor and Mutthulakshmi, 2024; Cerezo et al., 2024). Review scheduling introduces an additional requirement: the system must assign a delay before a knowledge component returns. Learner-state estimation and review timing draw on the same interaction history, but they produce different outputs—an estimate of what is currently known and an interval until the next encounter.

Review timing is especially complex in mathematics because correctness depends on more than access to an isolated memory trace. A knowledge component can recur across routine calculation, word problems, geometric reasoning, and procedures that depend on prerequisite skills. Repeated attempts can also reflect procedural practice, feedback-driven correction, or retrieval after a delay. Spaced and interleaved practice improves discrimination, transfer, and durable access to mathematical procedures and concepts (Rohrer and Taylor, 2007; Carpenter et al., 2012; Lindquist et al., 2024; Bego et al., 2024; Murray et al., 2025), yet the intervals recorded by a platform combine these different forms of practice within a single event stream.

Memory and spacing research provides the theoretical basis for separating them. Retention changes with elapsed time, and exponential, power-law, and hyperbolic curves offer different accounts of that change (Ebbinghaus, 1885; Wixted and Ebbesen, 1991; Rubin and Wenzel, 1996; Brown et al., 2007). Retrieval practice further shows that later access depends on both the timing and history of retrieval opportunities (Cepeda et al., 2006, 2008, 2009; Roediger and Karpicke, 2006; Karpicke and Roediger, 2008; Roediger and Butler, 2011; Rawson and Dunlosky, 2011). In platform data, however, elapsed time can index immediate response recovery, within-session practice, or delayed retrieval. Estimating a forgetting parameter without distinguishing these time scales risks conflating processes with different implications for review.

Personalized scheduling research has translated retention and retrieval history into adaptive review policies (Pavlik and Anderson, 2005; Lindsey et al., 2014; Kang et al., 2014; Tabibian et al., 2019), while knowledge tracing has refined estimates of learner–component state and next-response probability (Corbett and Anderson, 1995; Pelánek, 2017; Abdelrahman et al., 2023; Bai et al., 2024; Sun et al., 2024; Zhang et al., 2024b; Huang et al., 2024; Wang et al., 2025; Luo et al., 2025; Yu et al., 2025; Tong and Ren, 2025). Rule-based systems such as SuperMemo 2 (Wozniak, 1990), response-time-based adaptive systems such as ARTS (Mettler et al., 2011), and model-based scheduling by Pavlik and Anderson (2008) illustrate complementary ways of using performance, response time, or memory-strength estimates. These approaches converge on adaptive timing, but they differ in the signal used to update the schedule and in whether the output is a rule, a state estimate, or an optimized interval. The unresolved issue for routine mathematics-platform logs is how to identify which observed encounters carry delayed-retrieval information before a retention-based interval is calculated.

Routine mathematics-platform logs make it possible to examine this problem under naturally occurring practice conditions because they preserve the order, outcome, and timing of repeated KC encounters. Three questions guide the analysis: How does short-interval repetition affect the estimated relation between elapsed time and correctness? After prior encounters, previous correctness, KC differences, and learner baseline response tendency are included in the same model, how stable and predictively useful is learner-level temporal variation? What review-time scale is implied when unadjusted learner-level decay summaries are converted to a common retention target? Using the Junyi Academy data, we compare retention curves, estimate practice-history-adjusted delay effects, examine conditional learner-specific time slopes, and evaluate those slopes on later responses. The contribution is an empirical account of how rapid repetition changes the temporal signal recovered from platform logs and an assessment of the evidence still required before model-implied intervals can support learner–component scheduling.

## 2 Related work

### 2.1 Forgetting and spaced retrieval

Forgetting-curve research represents retention as a time-dependent quantity, but alternative functions distribute change differently across the retention interval. Ebbinghaus (1885) established the experimental study of time-dependent forgetting, after which the central question shifted from whether retention declines to how that decline is best described. Exponential functions impose a constant proportional rate, whereas power-law and hyperbolic functions allow a sharper early loss followed by slower change. Wixted and Ebbesen (1991) made functional form an explicit empirical question, and later quantitative and theoretical accounts related curve fit to the sampled time range and the temporal scale used to represent accessibility (Rubin and Wenzel, 1996; Brown et al., 2007).

Spacing and retrieval research moves from describing retention loss to organizing the events that alter subsequent access. Cepeda et al. (2006) consolidated the distributed-practice literature through a quantitative synthesis, and later work related effective spacing to the interval over which knowledge must be retained (Cepeda et al., 2008, 2009). Retrieval success also changes later retention, so the consequences of practice depend on whether an encounter requires effortful retrieval and on how retrieval opportunities are distributed (Roediger and Karpicke, 2006; Karpicke and Roediger, 2008; Roediger and Butler, 2011). Alongside the amount of practice, durable learning reflects the joint organization of elapsed time, retrieval success, and repeated access (Rawson and Dunlosky, 2011). Platform records place these processes in the same event stream: a short gap may preserve feedback and problem context, whereas a longer gap may follow intervening activity and reduced retrieval support. The temporal meaning of a recorded interval depends on the practice context in which that interval occurs.

### 2.2 Spacing in mathematics learning

Mathematics learning adds procedural dependency and prerequisite structure to the spacing problem. A knowledge component may be expressed through different problem formats, embedded in a longer solution path, or supported by related skills, so a correct response can reflect fluent retrieval, procedural reconstruction, transfer, or feedback-assisted correction. Rohrer and Taylor (2007) brought spacing and interleaving into mathematics practice by examining the effects of shuffling problem types, thereby linking the timing of practice with the selection and application of solution procedures. Work on concept induction and diverse instructional materials complements this account by showing why distributed examples can support discrimination and transfer across tasks (Kornell and Bjork, 2008; Carpenter et al., 2012). These relations extend general evidence for spaced and retrieval practice, but they also make the effect of a delay dependent on what the learner must recognize, retrieve, and execute at the next encounter (Dunlosky et al., 2013; Kang, 2016).

Delayed mathematics performance has consequently been examined across different curricular levels and forms of practice. Lindquist et al. (2024) tested spaced recall of fundamental concepts in a post-high-school precalculus course, extending laboratory accounts of retention to a course in which concepts and procedures recur over time. Spaced examples in advanced mathematics and retrieval practice across introductory STEM courses broaden the evidence base beyond a single task format (Zhang et al., 2024c; Bego et al., 2024). Murray et al. (2025) synthesized the effectiveness of spacing and retrieval practice for mathematics learning across instructional conditions. Across these settings, elapsed time operates together with problem format, prerequisite knowledge, interleaving, and the support available during retrieval. Mathematics-platform correctness is therefore a task- and support-dependent outcome because the same KC may be retrieved under different task structures and levels of support.

### 2.3 Personalized review scheduling

Review-scheduling methods differ in the information used to set the next encounter. Fixed schedules assign a common interval, whereas performance- and mastery-based rules respond to recent success or an estimated level of knowledge. Retention-based approaches add spacing and retrieval history, allowing two learners with similar current performance to receive different review times when their predicted rates of forgetting differ. The SuperMemo 2 tradition (Wozniak, 1990) exemplifies a rule-based update of intervals from review outcomes, while ARTS uses response-adaptive practice to regulate exposure. Pavlik and Anderson (2005) formalized practice and forgetting within an activation-based account of the spacing effect, and Pavlik and Anderson (2008) made the scheduling objective explicit by using a model to compute practice intervals.

Personalized scheduling extends this logic by updating review decisions as an individual's retrieval history develops. Lindsey et al. (2014) translated learner-specific retention estimates into personalized review and evaluated their relation to long-term knowledge retention. Questions about schedule form remain relevant within that approach: expanding and equal-interval retrieval schedules organize the same practice opportunities differently over longer periods (Kang et al., 2014). Tabibian et al. (2019) advanced the progression from parameterized retention models to optimization-based scheduling, in which review times are selected under an explicit learning objective. The response-time work of Mettler et al. (2011) likewise treated latency as a useful adaptive signal, which differs from a decay parameter estimated from correctness across elapsed intervals. Across these traditions, recommendations depend on which encounters are treated as evidence of retention change. Immediate corrective attempts, same-session repetitions, and delayed retrievals can contribute different information to the estimated rate, making event selection central to learner-specific decay estimation and review-interval calculation.

### 2.4 Knowledge tracing and review timing

Knowledge tracing estimates how a learner's state changes through repeated interactions with a knowledge component. Corbett and Anderson (1995) introduced a probabilistic account of procedural knowledge acquisition in which observed responses update an unobserved mastery state. Bayesian and logistic formulations differ in their assumptions about state change and response generation, but both organize interaction histories around the probability of current or subsequent success. Pelánek (2017) situated these formulations within a broader family of learner models, clarifying the connections among Bayesian tracing, logistic models, and later extensions; subsequent surveys document the expansion toward deep and more heterogeneous representations (Abdelrahman et al., 2023).

Recent knowledge-tracing research separates several sources of complexity that were compressed in earlier state representations. Hierarchical targets, multiple-state representations, and mixture-of-experts architectures provide finer-grained accounts of knowledge and response behavior (Sun et al., 2024; Zhang et al., 2024b; Huang et al., 2024). Wang et al. (2025) explicitly modeled diversity in learning states, while long- and short-term graph representations distinguish information carried at different portions of the learner history (Yu et al., 2025). Forgetting-related state dependencies add temporal change to this richer representation of learning (Luo et al., 2025), and explainable knowledge tracing addresses how such state estimates can be inspected and interpreted (Bai et al., 2024). These developments extend the representation of what a learner is likely to know at a given opportunity; temporal variables typically enter as evidence used to update that estimate.

Learning-analytics systems place learner-state estimates within a wider set of educational decisions (Cerezo et al., 2024). Paulsen and Lindsay (2024) synthesized the development of learning-analytics dashboards toward closer attention to learning, while explainable analytics addresses how educational predictions can be inspected and used (Gunasekara and Saarela, 2025). Performance-prediction models provide a related signal by summarizing engagement and achievement patterns for instructional use (Fazil et al., 2024; Lyu et al., 2025). Tong and Ren (2025) connected cognitive and neural knowledge tracing with personalized path generation, complementing adaptive technologies and process-based learning-path methods (Essa et al., 2023; Zhang et al., 2024a). Review scheduling adds a distinct output requirement: it must express the learner's changing state as an elapsed time before the next opportunity. Forgetting-aware knowledge tracing and retention-based scheduling are therefore complementary; the former refines state estimation with temporal information, and the latter converts an estimated retention trajectory into an actionable review interval. In routine mathematics platforms, that conversion depends on separating the time associated with immediate practice from the time associated with delayed retrieval and on representing learner-specific rates of change.

## 3 Theoretical framework

### 3.1 Exponential retention model

Delayed correctness links a previous KC encounter to the timing of a subsequent response. For each revisit, the platform records whether the learner answered correctly and how much time had elapsed since the preceding encounter with that KC. Across repeated revisits, the relation between delay and correctness provides an empirical retention function. A scheduling rule can locate the delay at which the fitted probability reaches a chosen retention target.

For learner *i*, the exponential candidate is expressed in Equation 1 as follows:


$$\begin{array}{l} R_{i}\left(t\right)=R_{0}\exp\left(-k_{i}t\right), \end{array}$$ (1)

where *R*_0_ is the immediate-retention baseline and *k*_*i*_ is the learner-level rate of decline in delayed correctness under observed practice conditions. The baseline anchors the predicted probability near zero delay, and the decay parameter describes how quickly that probability changes with elapsed time. The exponential form follows memory-based scheduling models and provides a common parameterization for comparing learners across their observed KC revisits (Pavlik and Anderson, 2005; Lindsey et al., 2014; Tabibian et al., 2019).

Because the fit pools a learner's revisits across knowledge components, *k*_*i*_ is a descriptive learner-level summary of time-related change in delayed correctness. It does not estimate a separate retention trajectory for each learner–KC pair. The adjusted-slope sensitivity analysis tests whether learner-level variation remains when observed practice history, KC effects, and learner baseline response tendency enter the same model, and the chronological holdout analysis tests whether adding that variation improves prediction of later responses.

### 3.2 Target-retention rule

The scheduling rule uses a target retention level to convert the fitted curve into a delay. Let *R*_target_ be the predicted correctness accepted immediately before review. Solving *R*_0_exp(−*kt*) = *R*_target_ for the revisit interval gives the expression in Equation 2:


$$\begin{array}{l} t^{⋆}=\frac{1}{k}\log\left(\frac{R_{0}}{R_{\text{target}}}\right). \end{array}$$ (2)

At fixed *R*_0_ and target retention, *t*^⋆^ decreases as *k* increases, so the fitted curves imply different timing scales for learners with different rates of decline. The resulting interval is a mathematical consequence of the fitted exponential curve and the selected baseline and target. Its potential scheduling role depends on subsequent learner–KC estimation and prospective evidence that reviews delivered at the implied times achieve the intended retention.

## 4 Materials and methods

### 4.1 Data and sample

We used the publicly released Junyi Academy Online Learning Activity Dataset (Chen et al., 2020), which contains 16,217,311 problem attempts from 72,758 pseudonymized learner identifiers between 2018-08-01 07:45 UTC and 2019-08-01 00:00 UTC. A deterministic sample (seed 42) of 5,000 learners was drawn for analysis. Learner, exercise, problem, timestamp, and correctness fields were used to order events and define responses; the level-4 content label served as the operational knowledge-component (KC) unit. Optional time-spent metadata was not used.

Records missing an identifier, timestamp, KC label, or binary outcome were excluded, as were duplicate timestamps within a learner–problem history and non-positive intervals within a learner–KC history. Table 1 summarizes the resulting analytical sample. The full content log contains 25,785 problem identifiers, including 76 linked to two level-4 KCs through multiple content records; the analysis retained the operational level-4 label for each event and did not impose an external prerequisite graph. Figure 1 shows the distribution of level-3 nodes, level-4 knowledge components, and exercises across the level-2 content hierarchy.

**Table 1:** Analytical sample and platform-log data structure.

Quantity	Value
Learners	5,000
Problem attempts	1,091,614
Positive-delay revisits	125,564
Knowledge components	171
Exercises (ucid)	1,326
Problem identifiers (upid)	24,632
Observation window	Full release period (2018–2019)
Outcome variable	Binary correctness
Primary time unit	Days since learning or review

**Figure 1:**
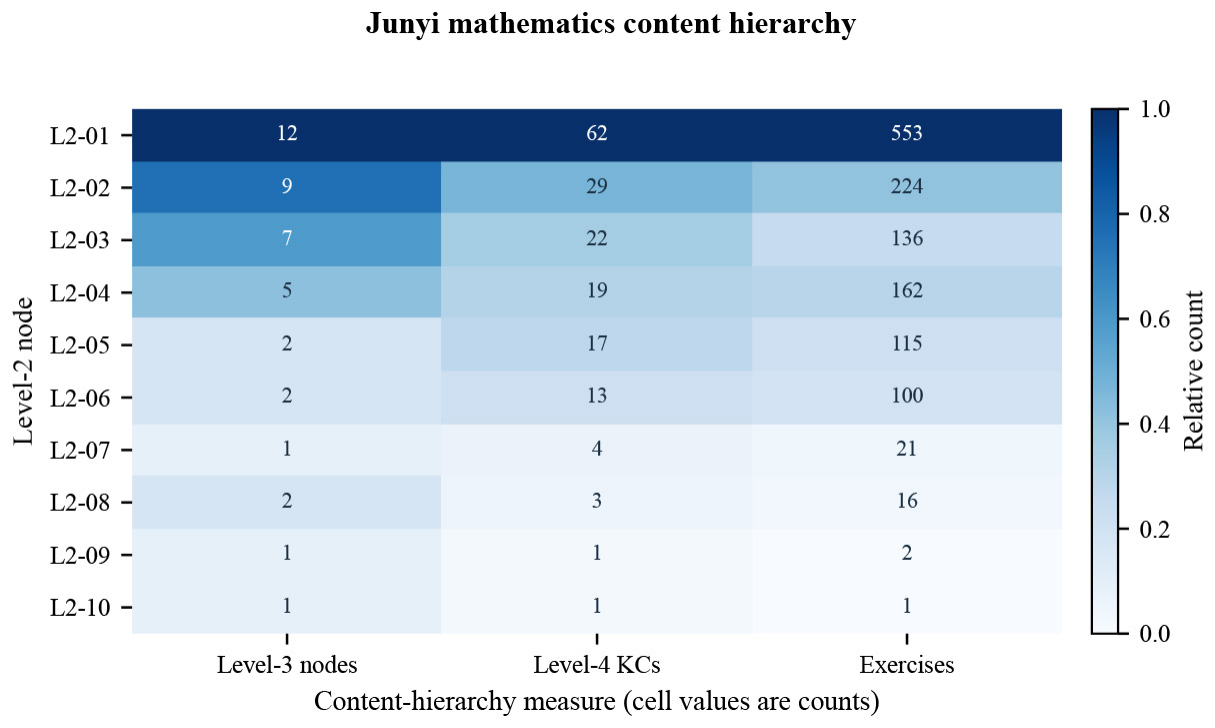
Junyi content hierarchy. The heatmap reports level-3 nodes, level-4 knowledge components, and exercises within each level-2 node; cell labels give the observed counts. The full log includes 76 problem identifiers linked to two level-4 knowledge components.

### 4.2 Revisit intervals and correctness

Within each learner–KC history, attempts were ordered by timestamp. For each revisit, the elapsed time was the fractional number of days since the immediately preceding attempt on that KC, and the outcome was the correctness of the later attempt. Positive-delay revisits were retained. The densest 0–14-day window was used for the primary comparisons, with empirical delayed correctness summarized in 20 logarithmically spaced bins after applying a 1-h minimum-delay rule. The pooled binomial generalized linear model (GLM) included elapsed time, log(1+ prior encounters), previous-attempt correctness, and 171 KC fixed effects; standard errors were clustered by learner.

The candidate curves were an exponential model, *R*(*t*) = *R*_0_exp(−*kt*), a power-law alternative, $R\left(t\right)=R_{0}{\left(1+t\right)}^{-\alpha }$, and a hyperbolic alternative, *R*(*t*) = *a*/(1+*bt*). *R*_0_ was constrained to [0.50, 0.99], and decay parameters were constrained to positive values. For each bin *b*, the fitted probability $\hat{R}\left(t_{b}\right)$ was compared with the observed number of correct responses *y*_*b*_ out of *n*_*b*_ attempts. The minimized objective was


-∑b[log(nbyb)+yblogR^(tb)+(nb-yb)log(1-R^(tb))]. (3)

Parameters were estimated by bounded numerical optimization. In the power-law model, α controls the exponent governing decelerating decay; in the hyperbolic model, *a* is the fitted immediate response level and *b* is the reciprocal-decay parameter. The likelihood uses *y*_*b*_ correct responses among *n*_*b*_ delayed responses in bin *b*, with $\hat{R}\left(t_{b}\right)$ evaluated at the median observed delay within each logarithmically spaced bin. Minimizing Equation (3) selects the parameter values that give the greatest likelihood to the observed correct and incorrect counts, with bins containing more observations contributing more strongly. The same aggregated binomial likelihood was used for all three alternatives before AIC and BIC were calculated. Additional analyses examined the delay-by-prior-practice interaction, adjusted learner-specific temporal slopes, alternative minimum-history thresholds, and robustness to problem identifiers linked to multiple level-4 KCs (Sections S1–S3 in Supplementary material and Sections S5–S9 in Supplementary material).

### 4.3 Learner-level estimation

The eight-revisit threshold means that at least eight eligible positive-delay revisit observations contributed to a learner-level fit. This coverage–precision rule provides a minimally informative history for a one-parameter likelihood estimate while retaining substantial learner coverage. The threshold was met by 2,173 learners; 65 histories had no variation in delayed correctness and were not estimable under the one-parameter likelihood, leaving 2,108 fitted learners for the descriptive learner-level analysis. Positive-delay revisits were pooled across each learner's KCs for a one-parameter fit with fixed *R*_0_ = 0.95. Decay-rate tertiles (703, 702, and 703 learners) were used to compare revisit counts and previous correctness. For the primary interval calculation, the estimator was refit after excluding gaps shorter than 1 h; the threshold sensitivity is reported in Supplementary Section S5.

Across the 4,241 learners with at least one positive-delay revisit, revisit counts had mean 29.61 (SD 64.10), minimum 1, Q1 2, median 8, Q3 28, 90th percentile 75, 95th percentile 128, 99th percentile 308.6, and maximum 1,489. There were 2,608 learners with at least five revisits and 2,173 with at least eight. These counts define the history-length distribution for learner-level estimation.

To examine immediate-retention heterogeneity, we also fit a two-parameter exponential model with learner-specific *R*_0*i*_ and *k*_*i*_ under the same 1-h and eight-revisit rules. Boundary counts and comparisons with the shared-baseline estimates are reported Section S4 in Supplementary material.

For the primary 1-h fit, 3,052 learners had at least one eligible revisit; 1,290 had at least eight, and 44 of those had no variation in delayed correctness and were not estimable. Among the 1,246 fitted learners, the number of observations per estimate had mean 41.64 (SD 56.32), Q1 12, median 21, Q3 47, and range 8–866. The threshold-specific refits in Supplementary Section S5 classify boundary status within each minimum-history sample; consequently, the eight-revisit refit has 1,114 interior estimates, compared with 1,110 in the prespecified primary summary.

Each learner-level *k* estimate was converted into a review interval at *R*_target_ = 0.80 using the shared immediate-retention baseline *R*_0_ = 0.95. A common baseline places learners on the same retention scale and avoids estimating two weakly identified parameters in histories with limited revisits. Sensitivity calculations for the baseline and learner-history threshold are reported Sections S4–S5 in Supplementary material. Interval summaries are given for the 1,110 interior estimates; the 130 lower-bound and 6 upper-bound fits are reported separately.

### 4.4 Conditional learner slopes and chronological validation

RQ2 was examined with a sparse L2-regularized logistic model fitted to the exact 1-h-to-14-day event sample of 48,833 revisits from 2,921 learners. The model included elapsed time, log prior encounters, previous-attempt correctness, level-4 KC indicators, learner intercepts, and learner-by-delay interactions. Continuous predictors were standardized for estimation and the learner-specific time slopes were returned to the per-day scale. We report the negative of each conditional log-odds slope as an adjusted decay slope, so larger positive values indicate a steeper decline. Two converged regularization settings (*C* = 0.1 and *C* = 1.0) were retained to assess the sensitivity of the high-dimensional learner effects; neither was designated as a uniquely preferred setting. Correlations with the unadjusted fixed-*R*_0_ estimates were calculated for the 1,112 learners estimable under both approaches.

Predictive value was evaluated with a chronological split among learners who had at least eight eligible 1-h revisits. For each of 1,155 learners, the earliest 80% of observations formed the training set and the latest 20% formed the test set, producing 34,669 training and 9,210 test rows. A base model containing elapsed time, practice history, previous correctness, KC indicators, and learner intercepts was compared with an extended model that added learner-by-delay interactions. Held-out log loss, Brier score, and area under the receiver-operating-characteristic curve were calculated at both regularization settings. A 2,000-replicate learner-cluster bootstrap provided the 95% confidence interval for the difference in log loss. Full specifications and results are reported in Supplementary Section S9.

## 5 Results

### 5.1 Revisit delay distribution

Revisit delays were concentrated at the shortest recorded interval but extended into multi-day retrieval. The median positive delay was 0.0104 days (15 min), whereas the third quartile was 1.99 days and the 90th percentile was 7.99 days. The short-gap concentration is consistent with within-session repetition and feedback-driven retry, while the upper tail contains delayed cross-session retrieval. After excluding gaps shorter than 1 h, 48,833 observations remained in the 20 log-spaced bins; exponential and hyperbolic curves were closely matched, whereas the power-law curve was less supported (Table 2).

**Table 2:** Candidate-curve comparison on 20 log-spaced delay bins after excluding gaps shorter than 1 h.

Model	Parameters	AIC	BIC	ΔAIC
Exponential	2	334.60	336.59	0.000
Power-law	2	352.67	354.66	18.068
Hyperbolic	2	334.66	336.65	0.057

AIC and BIC are computed from the aggregated binomial likelihood; Δ values are relative to the minimum.

The exponential and hyperbolic curves received nearly equivalent support over the 1-h analysis range, whereas the power-law curve was less consistent with the binned observations (Table 2). The exponential form was retained for the learner-level analysis because its single decay parameter with fixed *R*_0_ has a direct closed-form inversion to the target-retention interval and matches the parameterization used for the learner strata. This choice is a modeling convention for a common timing scale, not evidence that the exponential curve is uniquely superior; the near-tied hyperbolic fit is treated as model-form uncertainty and is discussed in Supplementary Section S8. Figure 2 shows the full delay distribution and the resulting analysis bands.

**Figure 2:**
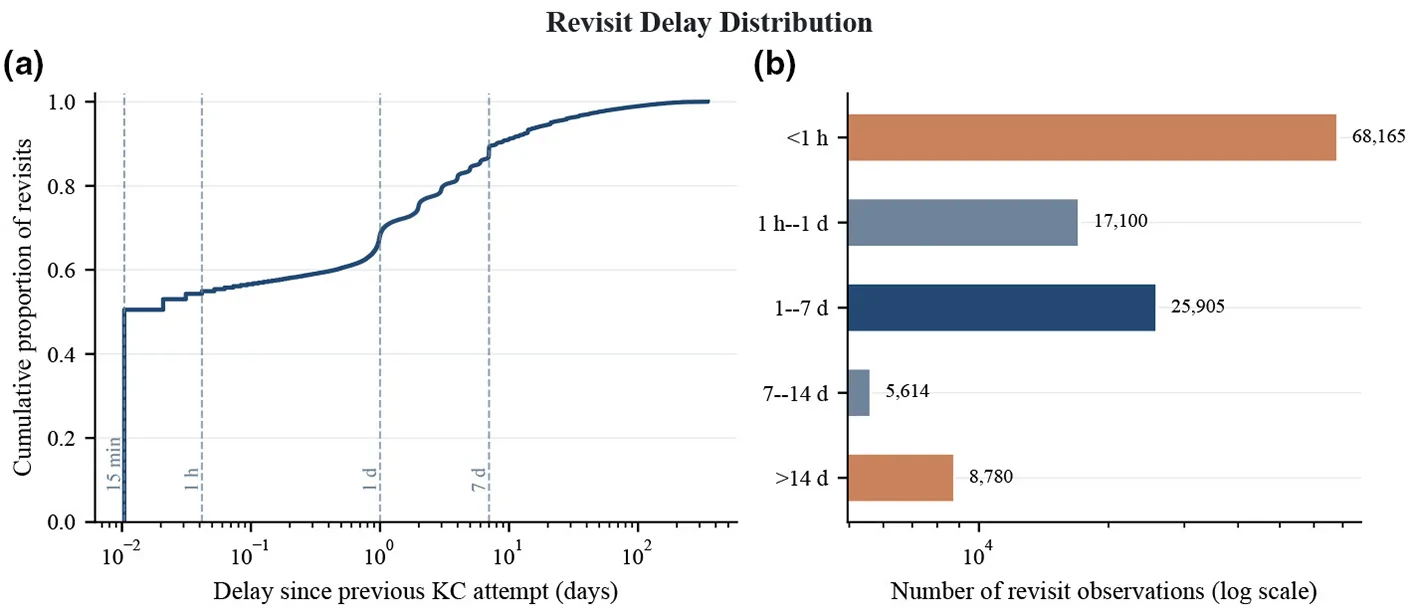
Revisit-delay distribution. **(a)** shows the empirical cumulative distribution of positive-delay revisits on a logarithmic delay axis; **(b)** shows counts in the delay bands used for the analysis.

### 5.2 Adjusted delay effects

When all positive delays were included, elapsed time contributed little additional information after practice history was entered. The 0–14-day model used 116,998 rows from 4,226 learners; its time coefficient was β = −0.00266 (clustered SE = 0.00299, odds ratio per day = 0.9973, *p* = 0.373). Previous correctness was strongly associated with later success (coefficient 0.602, SE = 0.0188, *p* < 10^−200^), whereas the log prior-encounter coefficient was 0.00661 (SE = 0.0104, *p* = 0.526).

The time effect became visible once the shortest repetitions were removed. At delays of at least 1 h, 48,833 rows from 2,921 learners gave β = −0.02098 (SE = 0.00383, odds ratio per day = 0.9792, *p* = 4.4 × 10^−8^); at delays of at least one day, 31,733 rows from 2,632 learners gave β = −0.02985 (SE = 0.00424, odds ratio per day = 0.9706, *p* = 1.9 × 10^−12^). The ordered change from 0.9973 to 0.9792 to 0.9706 indicates that including rapid repetitions changes the estimated magnitude of the time effect, not simply its precision (Figure 3).

**Figure 3:**
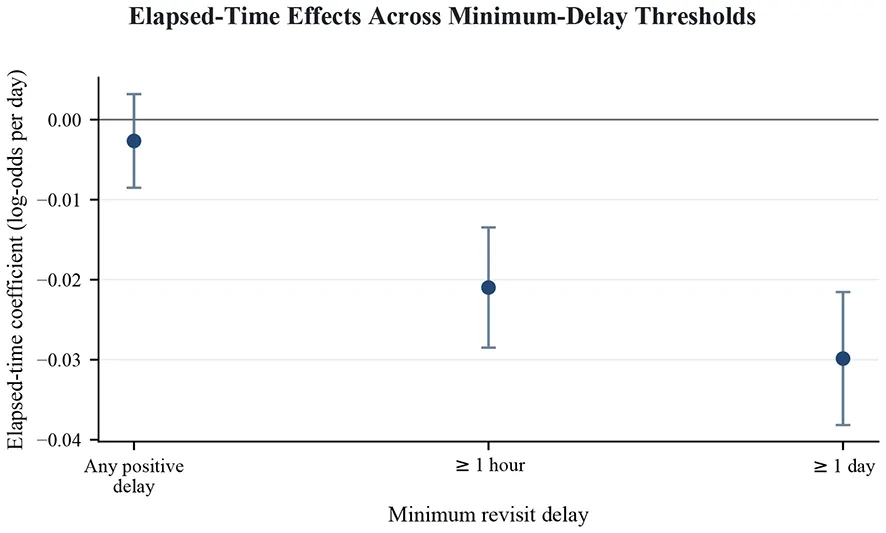
Practice-history-adjusted delay sensitivity. Points show pooled-binomial GLM coefficients and horizontal lines show 95% confidence intervals based on learner-clustered standard errors; KC fixed effects and prior-practice covariates are included.

The interaction between elapsed time and prior encounters was examined separately to determine whether the time effect varied across stages of repeated practice (Section S2 in Supplementary material and Supplementary Figure S1).

### 5.3 Learner-level decay estimates

Learner-level decay estimates covered a wide range and were not interchangeable with encounter count or previous correctness. Among 2,108 learners with at least eight positive-delay revisits, the median *k* was 0.0335 (Q1 = 0.0050; Q3 = 0.1662), with a small number of estimates at the upper bound of 5. The fastest-decay tertile had lower previous correctness (0.594) and fewer revisits on average (37.0) than the slowest-decay tertile (0.773 and 59.4), while mean prior-encounter counts were 31.5 and 30.0, respectively, with 34.4 in the middle tertile (Table 3).

**Table 3:** Learner strata formed from the fixed-*R*_0_ decay estimates.

Group	*n*	Mean *k*	SD *k*	Mean revisits	Mean previous correctness
Slowest decay	703	0.0027	0.0035	59.4	0.773
Middle tertile	702	0.0389	0.0225	72.9	0.684
Fastest decay	703	1.8778	2.1344	37.0	0.594

Revisit and previous-correctness summaries are descriptive.

The decay rate was negatively associated with revisit count (Spearman ρ = −0.169, *p* = 5.3 × 10^−15^) and mean previous correctness (ρ = −0.446, *p* = 9.1 × 10^−104^). The weaker association with revisit count and the stronger association with previous correctness indicate overlapping but non-equivalent information in practice exposure, recent success, and the fitted rate of decline. Figure 4 plots the learner distributions; the corresponding practice-history comparisons are reported Section S3 in Supplementary material and Supplementary Figure S2.

**Figure 4:**
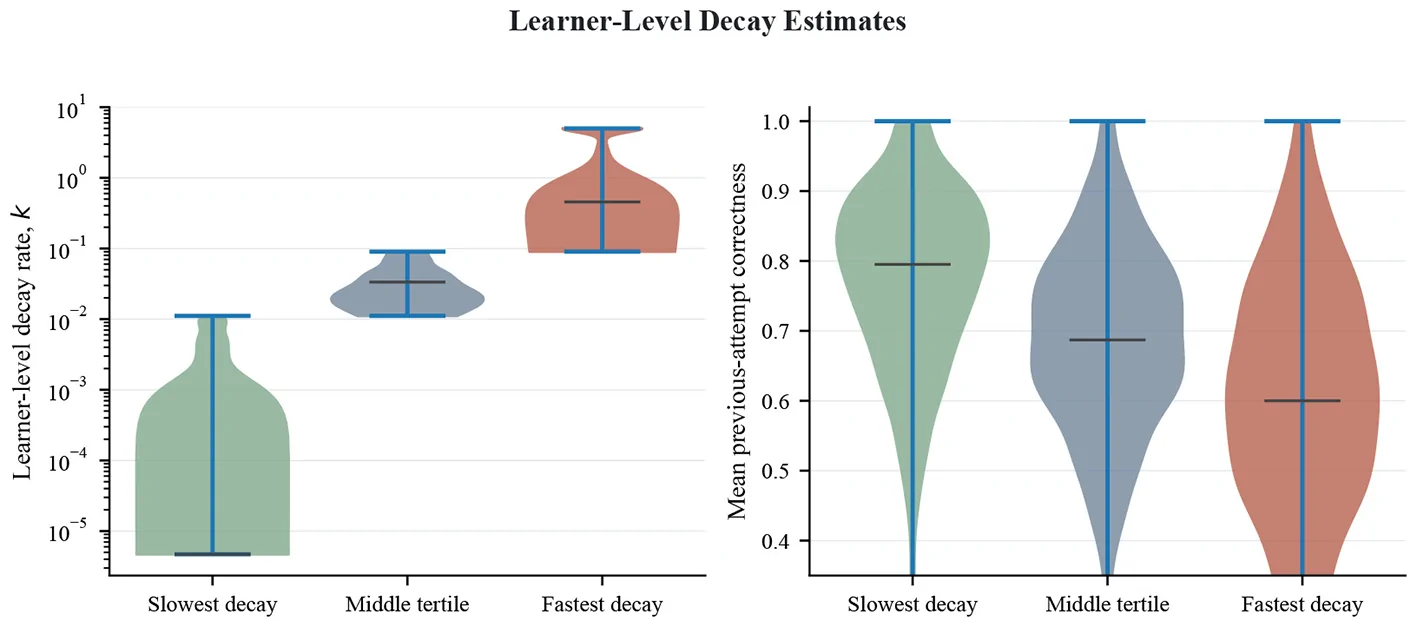
Learner-level decay and previous correctness across fixed-*R*_0_ decay-rate tertiles. Violin distributions show the fitted decay rate *k* (log scale) and mean previous-attempt correctness; central lines denote group medians.

### 5.4 Conditional learner-level temporal variation

Learner-specific time slopes remained dispersed after practice history, previous correctness, KC differences, and learner baseline response tendency entered the same model, but their estimated magnitude was sensitive to regularization. The exact event sample contained 48,833 1-h revisits from 2,921 learners; 1,112 learners also had an estimable unadjusted fixed-*R*_0_ comparison. At *C* = 0.1, the adjusted decay slopes had a median of 0.01534 per day and an IQR from −0.01219 to 0.04480. Relaxing the penalty to *C* = 1.0 widened the distribution substantially (median 0.00366; IQR −0.07526 to 0.08436).

The conditional slopes showed only weak correspondence with the unadjusted learner-level *k* estimates: Spearman correlations were ρ = 0.191 at *C* = 0.1 and ρ = 0.229 at *C* = 1.0. Once previous correctness was included in the same model, its learner-level association with the adjusted slopes was near zero (ρ = −0.019 and ρ = −0.017, respectively). Associations with revisit count (ρ = −0.084 and ρ = −0.054) and mean prior encounters (ρ = −0.049 and ρ = −0.037) were also small. Thus, adjustment removed the strong *post-hoc* association between the original *k* and previous correctness, but it did not reproduce the learner ordering obtained from the unadjusted exponential fits.

Adding learner-by-delay terms did not improve prediction for later responses in the chronological holdout sample. At *C* = 0.1, held-out log loss was 0.55688 for the base model and 0.55771 for the learner-slope model; the base-minus-slope difference was −0.000829 (95% learner-cluster bootstrap CI: −0.002282 to 0.000606). At *C* = 1.0, log loss increased from 0.55730 to 0.57030 after learner slopes were added, a difference of −0.012998 (95% CI: −0.017291 to −0.009136). Area under the curve also decreased at both settings. The adjusted slopes therefore document model-dependent conditional heterogeneity, but the holdout test does not support treating that heterogeneity as an incrementally predictive scheduling signal (Section S9 in Supplementary material and Supplementary Figure S4).

### 5.5 Target-retention intervals

The learner-level estimates produced widely different model-implied intervals at the same target retention. With *R*_0_ = 0.95 and *R*_target_ = 0.80, the 1-h fit produced a median interval of 6.21 days and an IQR of 2.12–16.66 days (Table 4). Of 1,246 fitted learners, 1,110 had interior *k* estimates; 130 were at the lower bound and 6 at the upper bound. The interquartile span covers more than two weeks, showing the heterogeneity implied by the shared-baseline exponential calculation. Because the adjusted learner slopes were weakly related to the unadjusted *k* estimates and added no holdout predictive value, this distribution describes the fitted model rather than validated learner–KC review times. As the fixed baseline *R*_0_ increased from 0.85 to 0.99, the median model-implied interval increased from 2.19 to 7.70 days (Table 5).

**Table 4:** Target-retention interval summary for the 1-h learner-level fit.

Statistic	Value
Eligible learners	1,246
Interior *k* estimates	1,110
Lower-bound *k* estimates	130
Upper-bound *k* estimates	6
Median interval (*R*_0_ = 0.95)	6.21 days
Interquartile range	2.12–16.66 days
Minimum–maximum (interior fits)	0.04–1,690.14 days
Share at or below 1.5 days (interior fits)	17.4%

Summary statistics are calculated among 1,110 interior *k* estimates; boundary fits are reported separately.

**Table 5:** *R*_0_ sensitivity for the 1-h learner-level fit.

*R*_0_	Median (days)	IQR (days)	Finite range (days)	Share ≤ 1.5 days
0.85	2.19	0.75–5.88	0.02–596.24	41.1%
0.90	4.26	1.45–11.42	0.03–1,158.39	25.9%
0.95	6.21	2.12–16.66	0.04–1,690.14	17.4%
0.99	7.70	2.63–20.65	0.05–2,095.77	13.5%

Medians, interquartile ranges, finite ranges, and shares are calculated among the 1,110 interior *k* estimates; 130 lower-bound and 6 upper-bound fits are retained in the total.

The joint *R*_0*i*_ – *k*_*i*_ sensitivity fit converged for all 1,246 learners but produced 139 lower-bound and 89 upper-bound *R*_0*i*_ estimates and 657 lower-bound *k*_*i*_ estimates. It yielded 502 finite intervals, with a median of 43.21 days. Joint-fit and fixed-baseline *k*_*i*_ estimates showed rank correspondence (Spearman ρ = 0.457, *n* = 1, 246, *p* = 1.8 × 10^−65^), which was stronger when both estimates were interior (ρ = 0.752, *n* = 586, *p* = 7.8 × 10^−108^). Absolute interval estimates consequently varied with the baseline specification.

Additional shared-baseline and learner-specific *R*_0_ sensitivity results are reported Section S4 in Supplementary material. Estimates were also recomputed under alternative minimum-history thresholds (Section S5 in Supplementary material and Supplementary Figure S3).

## 6 Discussion

The strengthening of the adjusted time effect after 1-h and one-day thresholds indicates that platform logs contain multiple temporal processes. Repetition after 15 min can preserve the problem context, corrective feedback, and a recently retrieved or reconstructed response. Such attempts often reflect same-session response recovery or error correction. Longer gaps introduce intervening activity and reduced immediate support, making later correctness more closely related to delayed retrieval and memory accessibility. This pattern connects the platform evidence with spacing and retrieval research, where the consequences of an encounter depend on both its timing and the conditions of retrieval (Cepeda et al., 2006, 2008; Roediger and Karpicke, 2006; Roediger and Butler, 2011; Rawson and Dunlosky, 2011). For learning-analytics models, pooling immediate retries with cross-session revisits compresses these processes into one delay coefficient and weakens the time relation needed for retention-based scheduling. The close exponential and hyperbolic fits after 1 h further suggest that model form is less consequential over this observed range than the definition of which encounters represent delayed retrieval.

Learner-level variation requires a more qualified interpretation. The unadjusted *k* estimates were most strongly associated with previous correctness (ρ = −0.446), whereas their associations with revisit count (ρ = −0.169) and mean prior encounters (ρ = −0.0440) were weaker. Entering previous correctness, prior encounters, KC indicators, and learner intercepts directly into the learner-slope model reduced the association between the adjusted slopes and previous correctness to approximately zero. This conditional separation did not carry over to stronger evidence of a stable learner timing trait: the adjusted slopes correlated only weakly with the original *k*, changed substantially with regularization, and failed to improve prediction for later responses. Statistical heterogeneity in a fitted time slope and incremental value for scheduling are therefore different evidential claims.

This distinction clarifies the relation between knowledge tracing and review timing. Knowledge-tracing models estimate mastery, readiness, or next-response probability from learner–component histories, and forgetting-aware variants can incorporate elapsed time directly (Corbett and Anderson, 1995; Pelánek, 2017; Abdelrahman et al., 2023; Bai et al., 2024; Sun et al., 2024; Zhang et al., 2024b; Huang et al., 2024; Wang et al., 2025; Luo et al., 2025). The exponential calculation used here performs a further transformation: it maps a pooled learner-level decay summary to the time at which a specified probability is reached. That mapping is interpretable, but one parameter pooled across KCs cannot represent differences in retention across a learner's algebraic, geometric, or procedural knowledge. Existing scheduling models that update component-specific state from performance and practice history (Pavlik and Anderson, 2005, 2008; Lindsey et al., 2014; Tabibian et al., 2019) consequently provide the appropriate context: a learner-level time summary would need to demonstrate incremental value within such a model, rather than replace it.

The interval distribution shows the scale of variation implied by the selected assumptions. With *R*_0_ = 0.95 and a target of 0.80, the median was 6.21 days and the middle half ranged from 2.12 to 16.66 days. The absolute values changed markedly when the common baseline changed, and jointly estimating *R*_0_ and *k* produced many boundary estimates. Exponential and hyperbolic curves also fit the population data almost equally well. These results make the interval calculation useful as a transparent analytical illustration of how a decay estimate can be expressed in days, while the sensitivity and holdout findings show why the resulting value is not yet an empirically established review time for a learner–KC pair.

The observational platform records leave several issues for subsequent research. The pooled learner-level fit combines elapsed time with item difficulty, feedback, sequence, study context, and unequal practice histories; level-4 KC tags incompletely represent prerequisite relations and problems that draw on multiple skills. Sparse histories contribute boundary estimates, and the shared baseline and functional form influence the interval scale. Most importantly, the analysis contains no intervention in which a specific KC is returned at its model-implied time. The next step is a learner–KC model that jointly represents current state, practice history, item and prerequisite structure, and time-related change, followed by prospective tests of calibration and delayed learning outcomes.

## 7 Conclusion

The Junyi logs establish that rapid repetition and delayed retrieval contribute differently to estimates of time-related decline: the adjusted daily effect strengthened systematically when 1-h and one-day minimum delays were imposed. Unadjusted learner-level fits varied widely and could be expressed as model-implied intervals, with a median of 6.21 days and a middle 50% of 2.12–16.66 days under the shared-baseline exponential specification. Yet learner-specific slopes estimated together with practice history, previous correctness, KC effects, and learner baselines were sensitive to regularization and did not improve prediction for later responses. The principal contribution is therefore to identify event selection as a central problem in recovering temporal information from mathematics-platform logs and to locate the interval conversion as an initial step, not a completed learner–KC scheduling method. Building on this evidence requires component-specific temporal estimation and prospective calibration, providing a focused path toward retention-sensitive, individualized review timing in adaptive mathematics practice.

## Data Availability

The original contributions presented in the study are included in the article/Supplementary material, further inquiries can be directed to the corresponding author.
