# Supplementary material for: Estimating time-related decline from mathematics platform logs: rapid repetition, learner variation, and model-implied review intervals
Source: Front Psychol. 2026 Oct 28;17:1908596. doi: 10.3389/fpsyg.2026.1908596 (PMC13752038; doi:10.3389/fpsyg.2026.1908596)
Supplement: Supplementary file 1 [file Data_Sheet_1.pdf]

Supplementary Material

SUPPLEMENTARY ANALYSES

S1 Practice-History-Adjusted Delay Model

For learner i, knowledge component c, and revisit j, the delayed outcome is $Y_{icj} \in \{0, 1\}$. Delay was calculated according to Equation (S1), where time is measured in days:

$$D_{icj} = \frac{T_{icj} - T_{ic,j-1}}{86400}. \tag{S1}$$

Prior practice was defined using Equation (S2), and previous correctness was defined using Equation (S3):

$$N_{icj} = j - 1. \tag{S2}$$

$$P_{icj} = Y_{ic,j-1}. \tag{S3}$$

The practice-history-adjusted GLM is given in Equation (S4):

$$\text{logit}[\Pr(Y_{icj} = 1)] = \beta_0 + \beta_1 D_{icj} + \beta_2 \log(1 + N_{icj}) + \beta_3 P_{icj} + \gamma_c, \tag{S4}$$

where γ_c denotes level-4 KC fixed effects. Standard errors are clustered by learner. The 0–14-day analysis retained all positive delays; the sensitivity specifications imposed minimum delays of one hour and one day.

Table S1. Practice-history-adjusted delay effects across minimum-delay specifications.

Minimum delay	Rows	Learners	Delay β	Clustered SE	OR/day	95% CI	p
Any positive delay	116,998	4,226	-0.00266	0.00299	0.9973	0.9915–1.0032	0.373
≥1 hour	48,833	2,921	-0.02098	0.00383	0.9792	0.9719–0.9866	4.4×10^{-8}
≥1 day	31,733	2,632	-0.02985	0.00424	0.9706	0.9626–0.9787	1.9×10^{-12}

For the unrestricted specification, the previous-correctness coefficient was 0.602 (SE 0.0188, $p < 10^{-200}$) and the prior-practice coefficient was 0.00661 (SE 0.0104, $p = 0.526$). At the one-day threshold, the corresponding coefficients were 0.636 (SE 0.0326, $p = 4.6 \times 10^{-85}$) and 0.0314 (SE 0.0159, $p = 0.049$).

S2 Delay-by-Practice Interaction

The interaction specification in Equation (S5) tests whether the delay effect changes with prior exposure:

$$\begin{aligned} \text{logit}[\Pr(Y_{icj} = 1)] = \beta_0 + \beta_1 D_{icj} + \beta_2 \log(1 + N_{icj}) + \beta_3 P_{icj} \\ + \beta_4 D_{icj} \log(1 + N_{icj}) + \gamma_c. \end{aligned} \tag{S5}$$

The interaction hypothesis is $H_0 : \beta_4 = 0$ (Equation (S6)).

$$H_0 : \beta_4 = 0. \tag{S6}$$

Table S2. Interaction between elapsed time and prior encounters.

Minimum delay	Delay β	Prior encounters β	Previous correctness β	Delay × prior encounters β	Clustered SE	95% CI (β)	OR	95% CI (OR)	p
Any positive delay	−0.02338	−0.00060	0.60138	0.00640	0.00259	0.00132–0.01148	1.0064	1.0013–1.0116	0.0136
≥1 hour	−0.03198	0.01128	0.62898	0.00333	0.00322	−0.00298–0.00964	1.0033	0.9970–1.0097	0.302
≥1 day	−0.03125	0.02947	0.63619	0.00042	0.00397	−0.00736–0.00820	1.0004	0.9927–1.0082	0.915

For any positive delay, the interaction estimate was $\beta_4 = 0.00640$ (SE $= 0.00259$, coefficient 95% CI 0.00132–0.01148; OR $= 1.0064$, 95% CI for the OR 1.0013–1.0116). The corresponding coefficient intervals were -0.00298–0.00964 for the one-hour threshold and -0.00736–0.00820 for the one-day threshold (Table S2). Figure S1 shows adjusted prediction curves at the 25th, 50th, and 75th percentiles of prior encounters. These values were 12, 29, and 60 for any positive delay; 14, 33, and 66 for the one-hour specification; and 13, 31, and 62 for the one-day specification. Shaded regions show 95% confidence intervals.

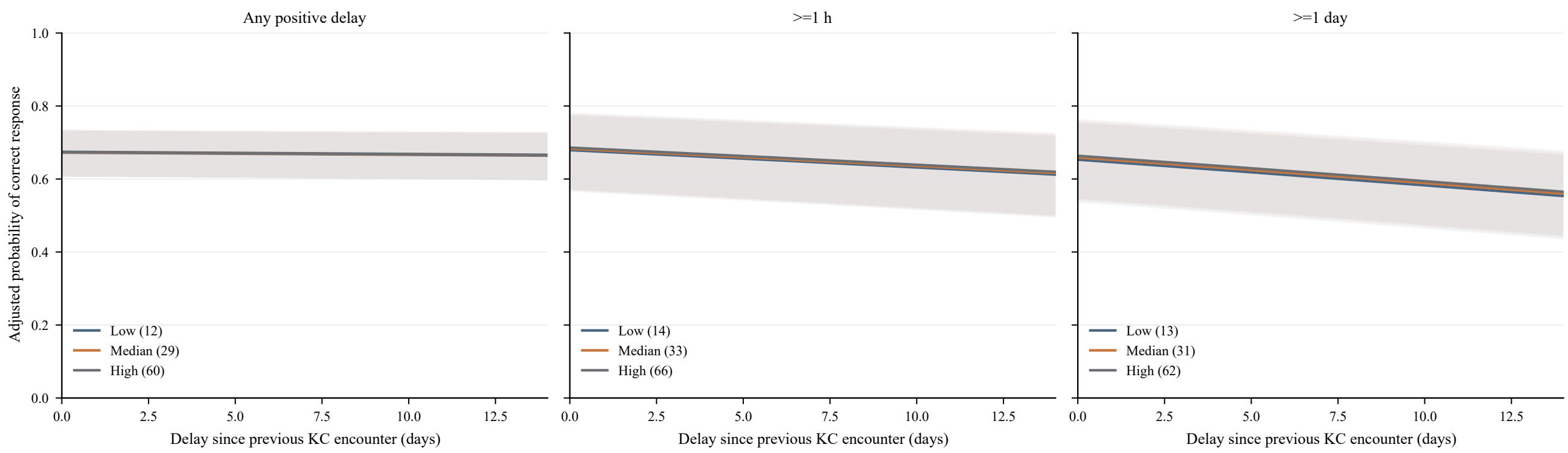

Figure S1. Adjusted delayed correctness across practice stages. Curves are shown for low, median, and high prior-practice levels defined by the empirical quartiles in each minimum-delay sample; ribbons denote 95% confidence intervals.

S3 Practice History by Decay Group

To quantify the relation between decay and observed practice history, we calculated the three Spearman correlations defined in Equations (S7)–(S9):

$$\rho_{k,N} = \rho\big(k_i, \overline{N}_i\big)\,, \tag{S7}$$

$$\rho_{k,M} = \rho(k_i, M_i)\,, \tag{S8}$$

$$\rho_{k,P} = \rho\big(k_i, \overline{P}_i\big)\,, \tag{S9}$$

where $\overline{N}_i$ is the learner's mean prior-encounter count, M_i is the learner's revisit count, and $\overline{P}_i$ is the learner's mean previous-attempt correctness. The resulting correlations were $\rho_{k,N} = -0.0440$ ($p = 0.0436$), $\rho_{k,M} = -0.1692$ ($p = 5.31 \times 10^{-15}$), and $\rho_{k,P} = -0.4464$ ($p = 9.14 \times 10^{-104}$). Overall differences among tertiles were assessed with Kruskal–Wallis tests for each history measure.

The Kruskal–Wallis statistics were $H = 197.14$ ($p = 1.55 \times 10^{-43}$) for revisit count, $H = 25.24$ ($p = 3.30 \times 10^{-6}$) for prior encounters, and $H = 399.79$ ($p = 1.54 \times 10^{-87}$) for previous correctness.

Table S3. Practice-history characteristics across learner decay tertiles.

Decay group	n	Median k	Mean k	Revisit mean	Revisit median [IQR]	Prior encounters mean	Prior encounters median [IQR]	Previous correctness
Slowest	703	0.000005	0.00269	59.41	29 [14–66.5]	29.95	26.6 [17.54–37.81]	0.773
Middle	702	0.03893	0.03893	72.90	41 [21–85.75]	34.37	28.58 [19.82–42.84]	0.684
Fastest	703	0.16046	1.87782	36.99	18 [11–36]	31.53	24.95 [15.28–38.10]	0.594

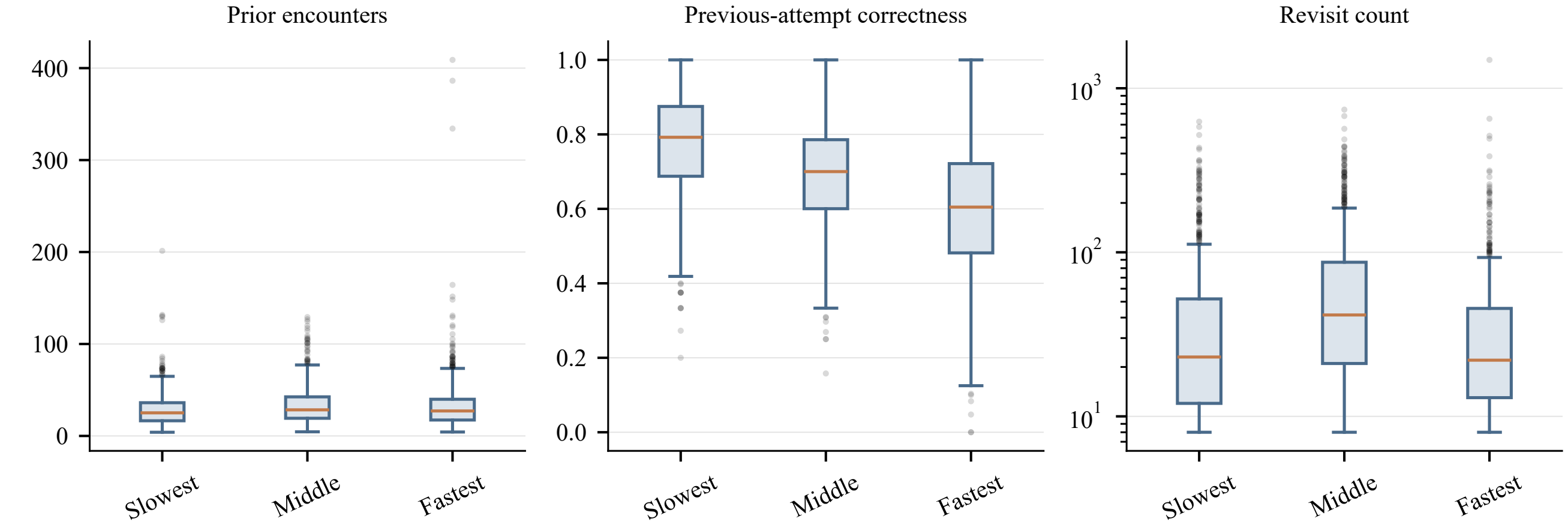

Figure S2. Practice history across decay-rate tertiles. Panels show prior encounters, previous-attempt correctness, and revisit count; points and box limits display the learner-level distributions used in Table S3.

S4 Shared and Learner-Specific R_0 Sensitivity

The learner-specific model in Equation (S10) is

$$R_i(t) = R_{0i} \exp(-k_i t), \tag{S10}$$

and target-retention intervals were calculated using Equation (S11):

$$t_i^* = \frac{1}{k_i} \ln\left(\frac{R_{0i}}{R_{\text{target}}}\right). \tag{S11}$$

Equations (S12) and (S13) describe the sensitivity of the interval to the shared baseline and the decay rate:

$$\frac{\partial t^*}{\partial R_0} = \frac{1}{kR_0} > 0, \tag{S12}$$

$$\frac{\partial t^*}{\partial k} = -\frac{\ln(R_0/R_{\text{target}})}{k^2} < 0. \tag{S13}$$

Thus a common R_0 changes interval magnitudes, whereas interval ordering at fixed R_0 is determined by k.

For the learner-specific R_{0i}–k_i fit, 1,246 learner histories were estimable. The fit reached the lower and upper R_0 bounds for 139 and 89 learners, respectively, and the lower and upper k bounds for 657 and 1 learners. There were 502 finite target-retention intervals, with median 43.21 days. Joint and fixed-baseline k estimates correlated at $\rho = 0.457$ ($p = 1.8 \times 10^{-65}$; $n = 1,246$) and $\rho = 0.752$ ($p = 7.8 \times 10^{-108}$; $n = 586$) when both estimates were interior.

Table S4. Shared-baseline and learner-specific R_0–k sensitivity analysis.

R_0	Median interval (days)	IQR (days)	Finite range (days)	Share ≤ 1.5 days
0.85	2.19	0.75–5.88	0.02–596.24	41.1%
0.90	4.26	1.45–11.42	0.03–1,158.39	25.9%
0.95	6.21	2.12–16.66	0.04–1,690.14	17.4%
0.99	7.70	2.63–20.65	0.05–2,095.77	13.5%

Learner-specific R_{0i}–k_i fit<br>Quantity	Value
Joint fits	1,246
R_0 lower-bound fits	139
R_0 upper-bound fits	89
k lower-bound fits	657
k upper-bound fits	1
Finite intervals	502
Median finite interval	43.21 days
Spearman ρ (all fits)	0.457
Spearman ρ (interior fits)	0.752

S5 Learner-History Sensitivity

Minimum-history sensitivity was assessed under the one-hour minimum-delay condition. The k-ranking correlations were calculated as defined in Equations (S14) and (S15):

$$\rho_{8,12} = \rho\left(k_i^{(8)}, k_i^{(12)}\right), \tag{S14}$$

$$\rho_{8,20} = \rho\left(k_i^{(8)}, k_i^{(20)}\right). \tag{S15}$$

The resulting correlations were $\rho_{8,12} = 1.000$ ($n = 983$) and $\rho_{8,20} = 1.000$ ($n = 677$). Intervals and k summaries in Table S5 use the converged likelihood fits at each threshold; boundary fits are included in the estimable count and percentage, whereas interval summaries are based on the finite fitted intervals.

Table S5. Sensitivity of learner-level estimates to minimum history length.

Minimum revisits	Eligible	Estimable	Interior	Median k	IQR k	Boundary fit	Median interval	IQR interval
5	1,673	1,551	1,378	0.032493	0.01101–0.08734	11.15%	5.29	1.97–15.61
8	1,290	1,246	1,114	0.026669	0.00999–0.07585	10.59%	6.44	2.12–16.66
12	999	983	883	0.023666	0.00933–0.06908	10.17%	7.26	2.49–18.42
20	681	677	616	0.020838	0.00827–0.05862	9.01%	8.25	2.93–20.78

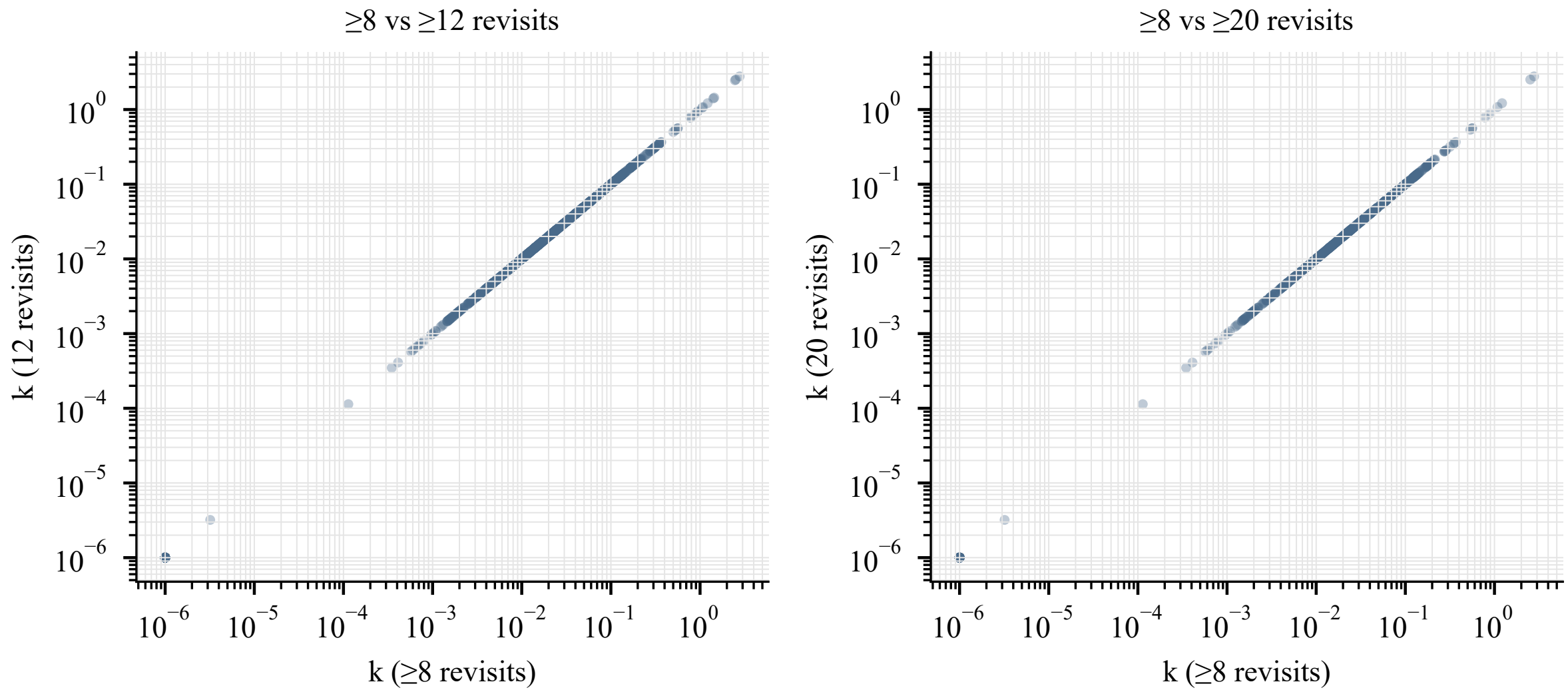

Figure S3. Stability of learner-level decay estimates across minimum-history thresholds. Scatterplots compare common learners under the eight- versus twelve-revisit and eight- versus twenty-revisit rules.

S6 Multi-KC Exclusion Analysis

The full content mapping contains 25,785 problem identifiers, of which 76 (0.295%) link to two level-4 KCs. In the analytical sample, 72 of 24,632 problem identifiers (0.292%) were observed with more than one level-4 KC; these identifiers contributed 6,984 of 1,091,614 attempts (0.640%). Excluding them produced the robustness estimates in Table S6.

Table S6. Delay-effect estimates after excluding multi-KC problem identifiers.

Sample	Minimum delay	Rows	Learners	Delay β	Clustered SE	OR/day	95% CI; p
Full sample	Any positive	116,998	4,226	-0.00266	0.00299	0.9973	0.9915–1.0032; 0.373
Excluding multi-KC IDs	Any positive	116,203	4,224	-0.00295	0.00301	0.9971	0.9912–1.0030; 0.327
Full sample	≥1 hour	48,833	2,921	-0.02098	0.00383	0.9792	0.9719–0.9866; 4.4×10^{-8}
Excluding multi-KC IDs	≥1 hour	48,431	2,921	-0.02122	0.00384	0.9790	0.9717–0.9864; 3.3×10^{-8}
Full sample	≥1 day	31,733	2,632	-0.02985	0.00424	0.9706	0.9626–0.9787; 1.9×10^{-12}
Excluding multi-KC IDs	≥1 day	31,483	2,631	-0.03030	0.00428	0.9702	0.9621–0.9783; 1.4×10^{-12}

S7 Curve-Fitting Likelihood and Information Criteria

For delay bin b, n_b is the number of observations, y_b is the number of correct responses, and t_b is the median observed delay within that bin. The 20 bins were defined by logarithmically spaced edges from 1 hour ($1/24$ day) to 14 days; thus, t_b is computed from the observed delays after bin assignment rather than from an arithmetic or geometric midpoint. The candidate probability is $p_b(\theta) = R(t_b; \theta)$. The corresponding binomial likelihood is given in Equation (S16). Candidate-curve parameters were estimated by minimizing Equation (S17), with the minimizer defined in Equation (S18) and AIC and BIC calculated using Equations (S19) and (S20):

$$L(\theta) = \prod_{b=1}^{B} \binom{n_b}{y_b} p_b(\theta)^{y_b} [1 - p_b(\theta)]^{n_b - y_b}, \tag{S16}$$

$$-\log L(\theta) = -\sum_{b=1}^{B} \left[\log \binom{n_b}{y_b} + y_b \log p_b(\theta) + (n_b - y_b) \log(1 - p_b(\theta)) \right], \tag{S17}$$

$$\hat{\theta} = \arg\min_{\theta} [-\log L(\theta)], \tag{S18}$$

$$\mathrm{AIC} = 2q - 2 \log L(\hat{\theta}), \tag{S19}$$

$$\mathrm{BIC} = q \log B - 2 \log L(\hat{\theta}), \tag{S20}$$

where q is the number of free curve parameters and $B = 20$ is the number of delay bins used for model comparison.

S8 Model-Form Sensitivity for Target-Retention Intervals

The learner-level interval summaries use the exponential parameterization because the fixed-baseline fit yields one decay parameter per learner and can be inverted directly. For comparison, the corresponding inversions for the alternative curves are

$$t^*_{\mathrm{power}} = \left(\frac{R_0}{R_{\mathrm{target}}} \right)^{1/\alpha} - 1, \tag{S21}$$

and

$$t^*_{\mathrm{hyperbolic}} = \frac{a/R_{\mathrm{target}} - 1}{b}. \tag{S22}$$

The one-hour population comparison gave AIC values of 334.60 for the exponential curve, 334.66 for the hyperbolic curve, and 352.67 for the power-law curve. Thus, the exponential and hyperbolic descriptions are empirically near-tied over the observed delay range. The reported 6.21-day median is consequently conditional on the exponential timing parameterization; the global fit does not establish a unique functional form. Equations (S21) and (S22) show how an alternative curve would be translated into a target- retention interval after its parameters had been estimated on the same data.

S9 Conditional Learner-Level Temporal Variation and Chronological Validation

Conditional learner-level time slopes were estimated from the exact one-hour-to-14-day event sample of 48,833 revisits from 2,921 learners. The sparse L2-regularized logistic model included standardized elapsed time and log prior encounters, previous-attempt correctness, level-4 KC indicators, learner intercepts, and learner-by-delay interactions. The adjusted decay slope is the negative of the common time coefficient plus the learner's time interaction, returned to the per-day log-odds scale. L2 regularization was needed for the high-dimensional learner effects, so both converged settings ($C = 0.1$ and $C = 1.0$) are reported in Table S7.

Panel A compares the 1,112 learners with estimable conditional slopes and unadjusted fixed-R_0 estimates. At $C = 0.1$, the association with unadjusted k was $\rho = 0.191$ ($p = 1.21 \times 10^{-10}$), whereas the associations with previous correctness ($\rho = -0.019$, $p = 0.519$), current correctness ($\rho = -0.039$, $p = 0.195$), and mean prior encounters ($\rho = -0.049$, $p = 0.102$) were small. The corresponding values at $C = 1.0$ were $\rho = 0.229$ ($p = 1.06 \times 10^{-14}$), $\rho = -0.017$ ($p = 0.565$), $\rho = -0.034$ ($p = 0.254$), and $\rho = -0.037$ ($p = 0.212$). The wider slope distribution at $C = 1.0$ shows that the estimated heterogeneity depends on the regularization applied to sparse learner histories.

Table S7. Conditional learner-level time-slope sensitivity and chronological holdout performance.

Panel A1. Conditional adjusted decay-slope summary

C	Rows	Learners	Comparison learners	Median	IQR
0.1	48,833	2,921	1,112	0.01534	−0.01219–0.04480
1.0	48,833	2,921	1,112	0.00366	−0.07526–0.08436

Panel A2. Spearman correlations with adjusted decay slopes

C	Unadjusted k	Previous correctness	Current correctness	Revisit count	Prior encounters
0.1	0.191	−0.019	−0.039	−0.084	−0.049
1.0	0.229	−0.017	−0.034	−0.054	−0.037

Panel B. Chronological holdout performance

C	Train rows	Test rows	Learners	Base log loss	Slope log loss	Base minus slope (95% CI)	Base AUC	Slope AUC
0.1	34,669	9,210	1,155	0.55688	0.55771	−0.000829 (−0.002282, 0.000606)	0.69159	0.68739
1.0	34,669	9,210	1,155	0.55730	0.57030	−0.012998 (−0.017291, −0.009136)	0.69306	0.68032

For Panel B, the earliest 80% of each eligible learner's observations were used for training and the latest 20% for testing. The base model contained the same fixed predictors and learner intercepts; the extended model added learner-by-delay terms. Positive values of base-minus-slope log loss would favor the extended model. At $C = 0.1$, the 2,000-replicate learner-cluster bootstrap interval included zero. At $C = 1.0$, the interval was entirely negative, indicating worse held-out performance after the learner-specific slopes were added. Brier scores showed the same direction: 0.18784 versus 0.18815 at $C = 0.1$ and 0.18782 versus 0.19230 at $C = 1.0$ for the base and extended models, respectively.

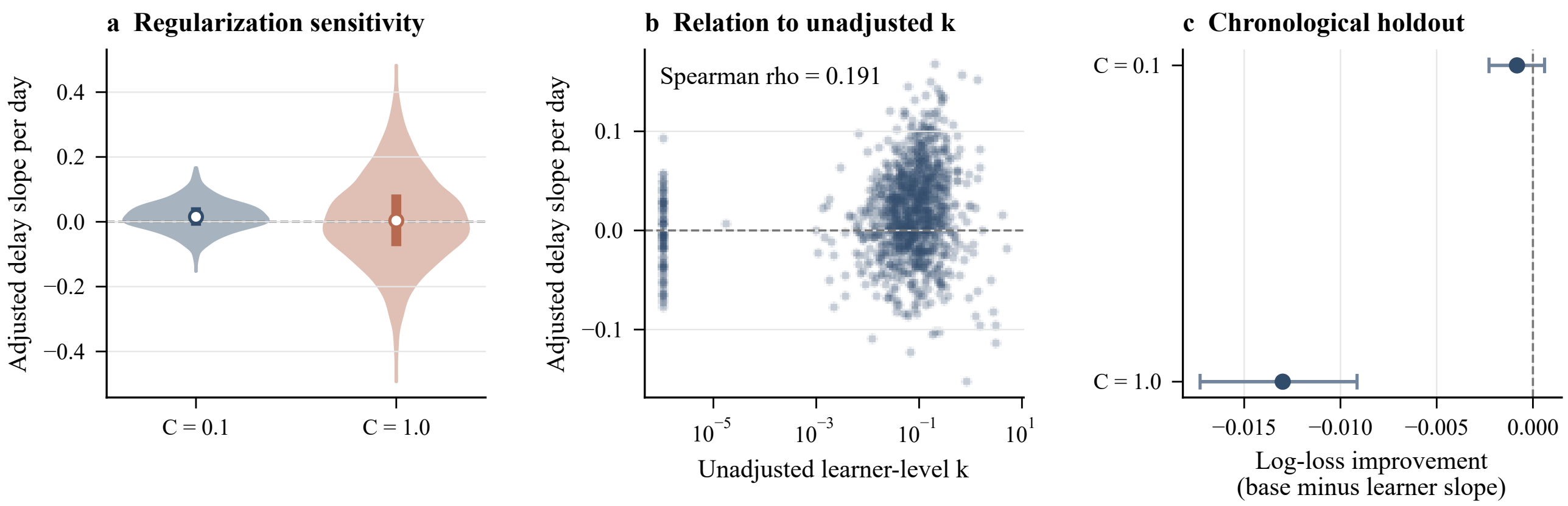

Figure S4. Conditional learner-level time-slope sensitivity and chronological validation. Panel (a) shows adjusted decay-slope distributions at both regularization settings; vertical bars span the interquartile range and open circles mark medians. Panel (b) compares the $C = 0.1$ adjusted slopes with the unadjusted fixed-R_0 estimates for 1,112 learners. Panel (c) reports the held-out log-loss difference between the base and learner-slope models, with 95% learner-cluster bootstrap intervals. Positive values favor the model with learner-specific slopes; neither setting improved held-out performance.
